# The Effects of Overweight and Obesity on Assisted Reproduction Technology Outcomes

**DOI:** 10.5935/1518-0557.20190005

**Published:** 2019

**Authors:** Rosa Lucia Silvestrim, Adriana Bos-Mikich, Marcos Iuri Roos Kulmann, Nilo Frantz

**Affiliations:** 1 Nilo Frantz Reproductive Medicine, Porto Alegre, RS, Brazil; 2 Basic Health Sciences Institute, ICBS – Federal University of Rio Grande do Sul, RS, Brazil

**Keywords:** obesity, overweight, polycystic ovarian syndrome

## Abstract

The aim of the present study was to assess the impact of professional nutrition assistance on assisted reproduction technology (ART) outcomes in overweight or obese patients with polycystic ovarian syndrome (PCOS). The study represents a retrospective analysis of fertilization rates, embryo quality and gestations after ART in seven PCOS patients, five obese and two overweight. The women attended a private Fertility Center in Brazil between the years 2010 and 2016. Out of the seven patients, the three that reached a successful gestation were the ones that underwent comprehensive lifestyle changes, taking care of their diet for a more prolonged period of time and reached an ideal weight loss during the nutrition counseling period.

## INTRODUCTION

Overweight and obesity are public health problems that are getting increasingly worse and have become a global epidemic according to the World Health Organization (WHO, 2011). Obesity is characterized by abnormal or excessive body fat accumulation to the point at which it becomes harmful to one’s health (WHO, 2016; Pinheiro *et al*., 2004). The energy imbalance between “energy intake” (food calories taken) and “energy output” (calories being used for body energy requirements), due to the intake of food that are low in nutrients and high in calories together with a sedentary lifestyle, is the leading cause of obesity (WHO, 2016). Endocrine disorders may take place due to an individual’s genetic background and due to a fattening environment, where low-nutrient, energy-dense food and drinks are frequently advertised influencing their consumption (Cruz Sanchez *et al*., 2013). The worldwide prevalence of obesity has more than doubled between 1980 e 2014.

According to the WHO (2016), in 2014, 1.9 billion adults aged 18 years or older were overweight, out of which, 600 millions were obese. The body mass index (BMI) is the worldwide index used to identify overweight and obesity in adults. Overweight is defined as a BMI equal to or greater than 25kg/m^2^, and obesity is when the BMI reaches 30kg/m^2^ or above (WHO, 2016). The higher the weight gain, the higher becomes the BMI and the likelihood of developing associated diseases, such as chronic non-transmissible diseases (CNTD), among which are breathing and cardiac problems, type II diabetes, hypertension and some types of cancer. In addition to the chronic diseases, weight excess may harm fertility due to hormonal disorders that affect the reproductive system (Becker *et al*., 2015). Overweight affects the reproductive physiology at different levels, including the hypothalamus (Tortoriello *et al*., 2004), the ovary and the ovarian follicle (Woodruff & Shea, 2011), the oocyte (Robker *et al*., 2009), the embryo (Jungheim *et al*., 2010) and the endometrium (Bellver *et al*., 2011). Considering that overweight is a frequent condition among women at reproductive age, it has already been associated with several fertility problems, such as anovulation, irregular menses, infertility, spontaneous abortions and gestational complications (Jungheim *et al*., 2013).

Obese women that seek assisted reproduction technologies for infertility treatment need higher doses of gonadotropins than normal weight patients to obtain adequate follicular development during ovarian stimulation cycles (Jungheim & Moley, 2010). Obese women present relatively poor oocyte quality and lower fertilization rates (Shah *et al*., 2011), they are less likely to achieve clinical pregnancy after IVF and embryo transfer (Jungheim *et al*., 2009), they have an increased risk of spontaneous abortion (Rittenberg *et al*., 2011) and are less likely to have a baby born after IVF (Luke *et al*., 2011). These situations may be caused by the poor oocyte and embryo quality found in obese patients (Metwally *et al*., 2007), and/or are due to an abnormal endometrial growth and embryo implantation (Jungheim & Moley, 2010).

Polycystic ovarian syndrome (PCOS) represents one of the most common endocrine disorders among women at reproductive age that may need assisted reproduction treatment to conceive (Dumesic *et al*., 2015). The prevalence of obesity among PCOS women ranges between 30 and 70% and it represents one of the several phenotypes associated with the syndrome (Pandey *et al*., 2010), together with risks related to the metabolic syndrome (Moran *et al*., 2010), to the non-alcoholic fatty liver disease (Baranova *et al*., 2011), to endocrine disruptors, particularly bisphenol A (Diamanti-Kandarakis *et al*., 2009), to cardiovascular diseases (Taponen *et al*., 2004), to insulin resistance (Norman *et al*., 2001) and to dyslipidemia (Valkenburg *et al*., 2008).

The metabolic alterations associated with PCOS, such as obesity, hyperinsulinemia, insulin resistance and a low-grade chronic inflammation, may all be important factors affecting oocyte competence and reproductive potential in this group of patients (Dumesic *et al*., 2015; Moran *et al*., 2015). Finally, it is important to mention that maternal obesity, regardless of PCOS status, may represent a risk factor to several congenital diseases in the offspring such as neural tube defects (Shaw *et al*., 1996), cardiac pathologies (Cai *et al*., 2014) and an increased likelihood of fetal death (Aune *et al*., 2014).

The objective of the present study was to evaluate the impact of professional nutrition assistance on assisted reproduction treatments, in terms of fertilization rates, embryo quality and gestations, for overweight-obese PCOS women.

## MATERIAL AND METHODS

This study is a retrospective analysis of seven cases of PCOS, of which five were obese and two were overweight with borderline BMI (almost obese). The patients came to the Nilo Frantz Human Reproduction Center between the years 2010 and 2016.

The patients’ records were obtained from the Clinic database and from their nutrition records. The women were diagnosed with PCOS according to the criteria established by the European Society for Human Reproduction and Embryology and the American Society for Reproductive Medicine, ESHRE-ASRM 2003 PCOS criteria (Rotterdam ESHRE/ASRM-Sponsored PCOS Consensus Workshop Group, 2004).

### Assisted Reproduction Technologies (ART)

The patients underwent controlled ovarian stimulation for ICSI. We employed the classical ART protocols for superovulation, oocyte collection, fertilization, embryo culture and transfer for ICSI cycles. Surplus, good quality embryos (not transferred) were cryopreserved. The parameters analyzed were total number of oocytes, day-2 and day-3 embryo quality (% of embryos grade 1 and 2 at day 2 or 3), day 5 or 6 blastocyst rate, biochemical gestations, abortions and live births.

### Nutritional follow-up

All seven patients were seen by the dietitian. The treatment was geared to the patients’ weight loss, lifestyle improvement, identification of their dietary pattern and promotion of changes in it to benefit the ART outcomes.

Initially weight and height were measured to calculate the BMI. Their weight was subsequently measured once a month. A nutritional anamnesis was performed to identify the pattern of food consumption, the need of possible changes and the introduction of new foods and eating habits.

Patients’ reports demonstrated that all seven women had similar eating patterns. They preferred carbohydrates, mainly refined, such as sugars, sweets and sugary drinks. The women reported continuous consumption, almost daily, of white bread, biscuits, cereal bars, pasta, pizza, margarine, ready-made fruit juices, soft drinks and yogurts containing sugar.

Based on this information, nutritional guidance was the same for all seven patients. They were recommended daily intake of vegetables, fruits, nuts and chestnuts, extra-virgin olive oil and fish. At the same time, it was suggested they decreased the consumption of red meat and the amount of carbohydrates, favoring the use of whole grain, refrain from refined carbs and soft drinks and keeping hydrated by drinking water. In addition, it was recommended they did not consume hot drinks in plastic cups or put plastic containers in the freezer, take Omega-3 supplements and get started or keep physical activities.

## RESULTS

The assisted reproduction centre where the study was performed registered a linear increase in overweight, obese I and obese II patients from 17% to 25%, from 5.5% to 6.5% and from 0.98% to 1.30%, respectively, between 2012 and 2016.

Only three out of the seven women that underwent ART had a pregnancy and live births. One of the pregnancies was a spontaneous gestation. The remaining four patients did not have a successful outcome from their ART treatments (Table 1). The three patients that achieved gestation and live birth were patients who adhered to the behavioral changes recommended by the dietitian.

**Table 1:** Physical characteristics and ART outcome of seven overweight/obese patients

Patient	IMC	Classif.	(% lost)	ART procedure	Age (years)	Oocytes MII (n)	Fert. rate (%)	Embryo Quality rate (%)	D5-6 Blast +/-	ET (+/-)/ Day/Bl	Endo (mm)	Outcome
1	37.85	Obesity II	13.6%	ICSI	36	10	100	60	-	+/D3	10	Abortion
												Natural pregnancy/Birth
2	37.28	Obesity II	9.22%	ICSI	39	12	83.3	40	+	+/D2	11	β-HCG (-)
				FET	39	----	----	----	----	+/Bl	10	Abortion
3	32.82	Obesity I	3.47%	ICSI (1)	37	7	71.4	60	+	+/D2	8	Abortion
				FET (1)	38	----	----	----	----	+/Bl	7.3	β-HCG (-)
				ICSI (2)	38	13	84.6	58.3	+	-	N/A	N/A
				FET (2)	39	----	----	----	----	+/Bl	7.7	Biochem. gestation
4	31.60	Obesity I	5%	ICSI (1)	31	11	36.8	28.6	-	+/D3	----	Biochem. gestation
				ICSI (2)	31	5	100	100	+	+/D3	----	Birth
5	30.23	Obesity I	13.53%	ICSI	30	17	76.5	61.5	+	+/D3	9.5	Birth/Twins
6	29.82	Overweight	1.17%	IUI (6)	38	----	----	----	----	----	----	β-HCG (-)
7	29.50	Overweight	2.9%	ICSI (1)	36	9	88	62.5	+	+/D3	7.8	Abortion
				FET (1)	37	----	----	----	----	+/Bl	6	β-HCG (-)
				ICSI (2)	38	11	72.7	62.5	+	+/D3	8	β-HCG (-)
				FET (2)	38	----	----	----	----	+/Bl	7.5	β-HCG (-)
				ICSI (3)	39	10	70	75	+	+/D3	7.5	β-HCG (-)
				FET (3)	39	----	----	----	----	+/Bl	8	β-HCG (-)

All seven patients presented a reasonable good number of MII oocytes collected, fertilization rates and embryo quality, regardless of their overweight/obese status. However, in the case of Patient 4 a significant improvement in embryo quality was detected in her second ICSI cycle, after she had started the nutrition therapy and changed her lifestyle.

## DISCUSSION

The three PCOS overweight or obese patients that reached gestation and live births were women that adhered to the diet and lifestyle changes recommended by the dietitian. These patients underwent lifestyle behavioral changes for a longer period and they presented a higher weight loss percentage during the period of nutritional follow up, than the patients who did not achieve gestation and live births. Patient 1 got pregnant naturally after she had an unsuccessful ART cycle and 18 months after she adhered to the treatment diet. It is likely that the new lifestyle and diet she adopted helped her to conceive. Unfortunately, this is a single case in the present study, and it is difficult to draw any conclusion from it. However, a study performed in Spain presented at the annual meeting of the European Society of Human Reproduction and Embryology (ESHRE) in Barcelona (2008), showed that natural conception may occur in infertile women who had previous ART cycle, because they maintain a healthy lifestyle. In addition, the study showed that excessive coffee and alcohol consumption and being significantly overweight reduces the likelihood of a subsequent natural conception.

On the other hand, patient 4, who underwent one ICSI cycle prior to the start of the nutrition therapy and another after adherence to the new routines and nutritional attitudes showed a remarkable improvement in embryo quality and achieved gestation and live birth. This observation is in accordance with previous reports that describe that embryo quality is associated with life style changes, which seem to influence term gestation and live birth (Metwally *et al*., 2007; Shah *et al*., 2011).

Endometrial growth, a key factor for successful term gestation did not seem to be affected by overweight or obesity. Except for one occasion, namely the second cycle of patient 7, all patients presented adequate endometrial thickness (>7mm) on the day of embryo transfer. On the other hand, there is controversy in the literature on whether obesity affects endometrial receptivity. A recent study by Coyne *et al*. (2016) on egg donation cycles for obese patients showed that embryos created using oocytes from healthy weight donors have implantation and pregnancy rates in obese recipients similar to those receipts with normal BMI. However, a previous study found opposite results (Bellver *et al*., 2013), showing that obesity reduces pregnancy rates in egg donation cycles, possibly by affecting endometrial receptivity.

It is relevant to notice that out of the four patients that underwent a significant weight loss, between four and 14Kg, after the initial nutritional counseling on dietary habits and lifestyle changes, three achieved full term gestations. The observed weight loss achieved between five and 18 months corresponds to a weight reduction between five and 13% of their initial values, which are in accordance with the expected weight loss for obese patients to get pregnant (Jungheim *et al*., 2009; 2013). In addition, the literature shows that obese women have an increased risk of having a spontaneous abortion, in addition to the fact that it takes longer to conceive when compared to women within the normal weight range (Rittenberg *et al*., 2011). Despite controversies, the British Fertility Society published guidelines in 2007, stating that severely obese women should have their fertility treatment deferred until they have lost weight. The recommendations were based upon a comprehensive analysis of studies which establish the adverse impact of obesity on fertility. The report, published in the BFS Journal of Human Fertility, targets clinics and specialists providing care to obese women before and during pregnancy (Balen & Anderson, 2007).

The negative effect of high BMI on the chances of PCOS patients becoming pregnant has been previously reported by Kulmann *et al*. (2016) on the same group of women investigated in the present study. The authors described lower positive beta-hCG and ongoing pregnancy rates among women with high BMI (>25), when compared to normal-weight women.

Overall, the present study shows that bad eating habits seem to be related to poor assisted reproduction outcomes, in terms of ongoing and full term gestations. On the other hand, positive ART outcomes may be achieved by women that adhere to the recommended nutritional and lifestyle changes and maintain the new habits for a period long enough to conceive and sustain a pregnancy.

It is important to point out that quick and short-term changes in weight loss may have negative effects on ART outcomes (Becker *et al*., 2015; Sim *et al*., 2014). It has been established that dietary and lifestyle changes need a minimum of three to five months to be effective for women to improve their odds of getting pregnant (Domecq *et al*., 2013; Moran *et al*., 2011; Islam *et al*., 2016; Juanola-Falgarona *et al*., 2014). Also, for example, the effects of one commonly used dietary supplement recommended to decrease inflammation in obese and overweight patients, occur at medium-long term consumption (Skulas-Ray *et al*., 2011).

The finding that no overweight or obese patient got pregnant after frozen embryo transfer may be explained by the hypothesis of putative higher lipid content in the blastomers of their embryos, which decreases post-thawing embryonic viability. It is well known among cattle breeders that donor cows, which are overweight or have a high-fat and high-calorie feed, present low quality oocytes and generate embryos with low post-cryopreservation survival rates, when compared with embryos from cows fed standard diets (Santos *et al*., 2008).

Despite the findings described in the present study and data from the literature, there is a lack of consensus on the negative effects of obesity on the female reproductive potential in ART cycles. Whereas some authors describe a decrease in conception likelihood due to obesity (Bellver *et al*., 2013), others did not find a relationship between BMI and pregnancy rates after ART (Shah *et al*., 2011). Thus, further studies involving a larger population of PCOS patients undergoing ART cycles are necessary to clarify the effects of obesity on conception and birth rates.
